# Public Sports Facility Availability in Living Communities and Mental Health of Older People in China: The Mediating Effect of Physical Activity and Life Satisfaction

**DOI:** 10.3390/bs15070991

**Published:** 2025-07-21

**Authors:** Shuhan Yan, Shengzhong Jiang, Xiaodong Dong, Xiuqi Guo, Mingzhe Chen

**Affiliations:** 1 School of Sociology, Nankai University, Tianjin 300350, China; yanshuhan@mail.nankai.edu.cn; 2 School of Finance, Nankai University, Tianjin 300350, China; jiangsz@nankai.edu.cn; 3 School of Public Health, Peking University, Beijing 100191, China; dongxd@hsc.pku.edu.cn; 4 China Center for Health Development Studies, Peking University, Beijing 100191, China; 5 School of Economics, Tianjin University of Commerce, Tianjin 300134, China; guoxiuqi@tjcu.edu.cn; 6 China Insurance and Social Security Research Center, Fudan University, Shanghai 200433, China

**Keywords:** public sports facility availability, mental health, living communities, aging, China

## Abstract

The aging of China’s population has created significant challenges for the mental health of older adults. However, limited research has examined how public sports facility availability in living communities supports older adults’ mental health. To explore this association, data were extracted from the 2016 China Longitudinal Aging Social Survey, which involved 7811 respondents. The ordinary least squares model and the instrumental variable approach were employed to test the association between public sports facility availability in Chinese older adults’ living communities and their mental health. The bootstrapping method was used to estimate the mediating effect of physical activity and life satisfaction. The results indicate that public sports facility availability in living communities was significantly correlated with a decrease in depressive symptoms among older people (coefficient = −0.225; *p* < 0.01), which suggests that a greater availability of public sports facilities in living communities is related to the better mental health of older adults. The results of the mediation analysis show that physical activity and life satisfaction were identified as mediating mechanisms. This study suggests that increasing the availability of public sports facilities in older adults’ living communities can alleviate depression and promote better mental health. Our findings provide valuable policy implications for enhancing public sports infrastructure and promoting healthy aging.

## 1. Introduction

In 2019, approximately one billion individuals worldwide were suffering from mental health disorders, with the prevalence of depression and anxiety increasing by 25% in 2020 (World Health Organization, 2022). Older adults are particularly vulnerable to mental health problems due to significant social and lifestyle transitions (Singh & Misra, 2009). In China, 26.4% of older adults exhibit depressive symptoms, with 6.2% experiencing moderate to severe depression (C. Y. Gao & Li, 2024). Mental disorders compromise not only psychological well-being but also physical health, significantly reducing quality of life (Luo et al., 2020) and increasing mortality risks, particularly through suicide (Lancet Global Mental Health Group, 2007). As the World Health Organization states, “there can be no health without mental health” (World Health Organization, 2005); therefore, identifying factors influencing mental health in older adults is crucial for its improvement.

China is facing a significant demographic shift towards an aging population, with the proportion of people aged 60 and above rising from 13.3% in 2010 to 22% in 2024 (National Bureau of Statistics of China, 2011, 2025). By 2050, this number is projected to reach approximately 487 million, representing almost one-third of the entire population (Xinhua News Agency, 2018). The rapid growth of the older population places increasing pressure on public health systems, heightening the urgency of addressing mental health concerns. Given the urgency of this public health challenge, researchers have sought to identify modifiable factors that may improve the mental health of older adults.

As mobility declines with age, older adults tend to remain predominantly within their local communities. Public sports facilities in living communities, as key community resources, can play a crucial role in supporting physical activity, socialization, and overall health in older adults. Despite the importance of public sports facilities, empirical research on the relationship between public sports facility availability in living communities and older adults’ mental health remains limited. This study aims to fill this gap by empirically examining the relationship between public sports facility availability in living communities and mental health among 7811 older adults from the 2016 China Longitudinal Aging Social Survey (CLASS). It further examines the mediating mechanisms of physical activity and life satisfaction within this relationship, alongside the moderating effects of age, income, education, and regional economic development. The remainder of this paper is organized as follows: Section 2 is a theoretical analysis. Section 3 details the data and methods. Section 4 reports the full range of empirical findings, from benchmark regressions to robustness checks and mechanism analyses. Section 5 discusses the broader implications of our results, and Section 6 concludes the study.

## 2. Theoretical Analysis

### 2.1. Public Sports Facility Availability in Living Communities and Older People’s Mental Health

Beyond individual-level factors, evidence underscores the significant influence of the built environment on the mental health of older adults. Factors like parks (Sturm & Cohen, 2014; Wood et al., 2017; Orstad et al., 2020), green spaces (Maas et al., 2009; Sokale et al., 2022), neighborhood walkability (Berke et al., 2007; Domenech-Abella et al., 2020), distance to facilities (Thomas et al., 2007), pavements (Araya et al., 2007; Mair et al., 2008), accessible services (Gong et al., 2016), and living environment (Weich et al., 2002; Saarloos et al., 2011; Rautio et al., 2018) all contribute to enhancing older adults’ mental health. As a key element of the built environment, public sports facilities in living communities may offer potential avenues for fostering social interaction and facilitating physical exercise. Recent studies have begun to recognize the role of community-level sports infrastructure and neighborhood recreational facilities in promoting residents’ well-being and decreasing older adults’ depressive symptoms (Mu et al., 2023; Wang et al., 2025), but research in this area often concentrates on broader green or recreational spaces, with specific attention to public sports facilities in living communities remaining comparatively limited.

Although public sports facilities are crucial for promoting health, the actual investment and facility provision remain insufficient. In 2023, China’s Culture, Tourism, Sports, and Media Expenditures accounted for CNY 396,536 million, which constitutes 14% of the overall public budget allocation (National Bureau of Statistics of China, 2024). The budget expenditure for basic public sports services is very limited. China’s per capita sports facility area is just 2.89 square meters, while in the United States and Japan, it exceeds 15 square meters (General Administration of Sport of China, 2015, 2023). There are a total of 28,137 parks nationwide (National Bureau of Statistics of China, 2024) and 608,000 urban and rural communities (Ministry of Civil Affairs of the People’s Republic of China, 2023). This means that each park is shared by 21.6 communities on average, making access to exercise spaces more difficult. Under such spatial distribution limitations, public sports facility availability in living communities serves as a critical compensatory mechanism for the scarcity of exercise spaces, thereby significantly enhancing opportunities for physical activity.

With advancing age, older adults experience a decline in mobility, which is characterized by a reduction in walking speed (Himann et al., 1988; Bohannon, 1997; Bohannon & Andrews, 2011) and an elevated risk of falls (Prince et al., 1997; Hausdorff et al., 2001; Kang & Dingwell, 2008). These age-associated mobility limitations can significantly magnify the adverse effects of inadequate public sports facilities. The theory of the Social Ecological Model points out that individual behavior is situated within a multi-tiered environmental system, and individual development is shaped not only by personal factors but also by the various levels of social environments in which the individual is embedded (Bronfenbrenner, 1979). According to the Social Ecological Model, macro-level deficits in spatial planning, such as a lack of neighborhood sports facilities, can interact with individual functional decline. Consequently, older adults may find themselves more confined to their homes, missing out on the physical and social activities that help maintain good mental health. Constraints in the built environment, such as inadequate public sports facilities, limit opportunities for health-enhancing behaviors, potentially exacerbating mental health disparities (Ortega et al., 2020).

Meanwhile, public sports facilities also function as symbols of age-inclusive urban planning. Their presence and accessibility may signal societal recognition of older adults’ right to health-promoting spaces, fostering a sense of belonging that counteracts depression-linked social exclusion (Lefebvre, 1991). Therefore, public sports facilities in living communities, as a vital component of the built environment and key community resource, hold considerable importance for older people and might directly shape health-related behavioral choices and mental health among them. Drawing upon the aforementioned discussion, we put forth the following hypothesis:

**Hypothesis 1.**  *Public sports facility availability in living communities is significantly and positively associated with better mental health among older individuals*.

### 2.2. The Mediating Roles of Physical Activity and Life Satisfaction

This study posits that public sports facility availability in living communities may influence older individuals’ mental health through physical activity and life satisfaction. First, evidence indicates that physical activities significantly promote better mental health among older people (O’Connor et al., 1993; Christmas & Andersen, 2000; Benedetti et al., 2008; Kandola et al., 2019; Pearce et al., 2022). Some earlier studies have indicated that public sports facilities are positively related to changes in physical activity (Kim & Kosma, 2013; Lee et al., 2016; W. Gao et al., 2022). Public sports facility availability in living communities creates opportunities to normalize exercise routines (Sallis et al., 2015). Indeed, the proximity of public sports facilities to the residences of older adults has been shown to significantly increase the likelihood of regular utilization (Addy et al., 2004). Furthermore, many older adults avoid using large- and medium-scale public sports facilities because they charge usage fees (W. Gao et al., 2022). Thereby, free and convenient access to public sports facilities can encourage older individuals to stay more physically active (Steenhuis et al., 2009).

Second, low life satisfaction has been linked to significant negative outcomes, particularly in mental and physical health (Huebner et al., 2006; Lin et al., 2024). Research suggests that life satisfaction plays a protective role in maintaining older adults’ mental health (Bray & Gunnell, 2006). Higher life satisfaction is generally linked to fewer depressive symptoms and better mental well-being in older adults (Headey et al., 1993; Soósová et al., 2021). Prior research suggests that the neighborhood built environment fosters social interaction and cohesion (Pfeiffer et al., 2020), both of which are key contributors to older people’s life satisfaction (Yu et al., 2019; Park & Kang, 2023). As a crucial component of the neighborhood built environment, public sports facilities can provide venues that facilitate social interaction and foster cohesion among individuals with shared interests in sports, ultimately enhancing life satisfaction (Moustakas, 2022).

Overall, considering the associations between public sports facility availability in living communities, physical activity, life satisfaction, and mental health, it can be hypothesized that physical activity and life satisfaction might function as mediating mechanisms linking public sports facility availability in living communities and mental health among older adults. Drawing upon the aforementioned discussion, we put forth the following hypotheses:

**Hypothesis 2a.**  *Physical activity mediates the association between public sports facility availability in living communities and older individuals’ mental health.*

**Hypothesis 2b.**  *Life satisfaction mediates the association between public sports facility availability in living communities and older individuals’ mental health.*

### 2.3. The Moderating Effect of Age, Income, Education, and Regional Economic Development

Age, income, education, and regional economic development may moderate the relationship between public sports facility availability in living communities and mental health in older adults. Compared to younger seniors, older seniors experience greater mobility limitations, leading to a decline in both the number and distance of their daily trips (Boschmann & Brady, 2013). These constraints reduce their ability to access distant areas for physical activities (Lord et al., 2011), underscoring the necessity of conveniently located exercise opportunities (Yen et al., 2009). Consequently, older seniors’ mental health might be more influenced by public sports facility availability in living communities.

Older people with a low income often face financial barriers to accessing paid sports venues, making free or low-cost public sports facilities their primary avenue for physical activity (Steenhuis et al., 2009; Withall et al., 2011). Additionally, limited financial resources restrict their opportunities for social participation, increasing their reliance on community-based activities to maintain social connections (Feng et al., 2020). Therefore, the mental health of older individuals with a low income might be more influenced by public sports facility availability in living communities.

Older adults with lower educational attainment tend to exhibit reduced literacy skills (Barrett & Riddell, 2019) and may have a more limited capacity to access, understand, and apply health-related knowledge (Howard et al., 2006). Their limited health knowledge and reduced health literacy make it more challenging to proactively seek health-related information (Friis et al., 2016). Consequently, they may be more reliant on tangible and easily accessible sports facilities to participate in physical activities. Therefore, the mental health of older individuals with lower educational attainment might be more influenced by public sports facility availability in living communities.

Regions with higher economic development invest greater resources in public infrastructure, thereby ensuring the quality and accessibility of facilities for older populations (Andreff, 2001; Burillo et al., 2011). In contrast, provinces with lower economic development and insufficient public infrastructure investment frequently experience a shortage of sports facilities, which can adversely affect residents’ opportunities for physical activity and subsequently their health (Pascual et al., 2007). Therefore, the availability and quality of public sports facilities in regions of high economic development may play a more significant role in shaping the mental health outcomes of older people living there. Drawing upon the aforementioned discussion, we put forth the following hypotheses:

**Hypothesis 3a.**  *With advancing age, the association between public sports facility availability in living communities and older individuals’ mental health is stronger.*

**Hypothesis 3b.**  *With lower educational attainment, the association between public sports facility availability in living communities and older individuals’ mental health is stronger.*

**Hypothesis 3c.**  *With lower income, the association between public sports facility availability in living communities and older individuals’ mental health is stronger.*

**Hypothesis 3d.**  *With advancing regional economic development, the association between public sports facility availability in living communities and older individuals’ mental health is stronger.*

## 3. Methods

### 3.1. Data

The data for this study were extracted from the China Longitudinal Aging Social Survey (CLASS), a nationally representative survey initiated by the Renmin University of China. For this study, we defined our sample as individuals aged 60 and over. This age threshold was chosen based on three considerations. First, it is consistent with the official definition of “older adults” in China’s national policies and strategic plans on aging (Standing Committee of the National People’s Congress, 2018; Ministry of Civil Affairs of the People’s Republic of China, 2024). Second, this criterion ensures comparability with the existing literature on aging in China (Chen et al., 2012; Liu et al., 2017). Finally, it is consistent with the design of the CLASS itself, which specifically targets the population aged 60 and above. Following the exclusion of outliers and missing data, the final analytical dataset consisted of 7811 individuals aged between 60 and 103 years.

### 3.2. Measures

#### 3.2.1. Explained Variable

This study focused on older individuals’ mental health as the explained variable. To measure mental health, this study employed the 9-item short version of the Center for Epidemiological Studies Depression (CES-D) scale, developed by Radloff (1977). The CES-D scale is extensively validated as a reliable instrument for measuring mental health (Jiang & Yang, 2022). The short version has been shown to exhibit comparable validity to the original 20-item version (Shrout & Yager, 1989). In the CLASS questionnaire, respondents were asked about their emotional states over the past week, with specific items based on the CES-D scale detailed in the Appendix A. The final mental health scores ranged from 0 to 18, with higher scores denoting greater depressive symptoms and worse mental health.

#### 3.2.2. Explanatory Variable

This study focused on public sports facility availability in living communities as the explanatory variable, defined as the adequacy and availability of free or public resources that met residents’ needs. Specifically, the measure of the explanatory variable was based on responses to the following question in the CLASS 2016: “Are there any of the following venues or facilities for activities in your community?” The questionnaire included three types of venues: senior activity rooms, fitness venues/facilities, and outdoor activity areas. The respondents were assigned a score of 1 if any of these venues were present in their communities and 0 if none were available. The final availability score was calculated by summing the available venue types, and a composite score ranging from 0 to 3 was derived.

**Figure 1:**
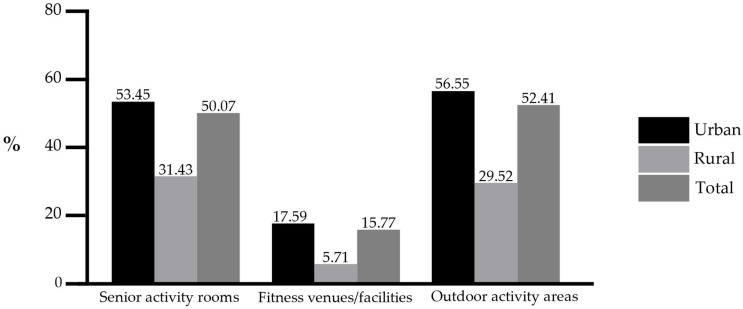
Proportion of older adults living in communities with different types of sports venues/facilities.

Figure 1 presents the overall availability of various physical activity venues and facilities in older people’s living communities. The findings indicated that public sports resources remained insufficient, limiting public sports facility availability for older adults. Notably, the proportion of fitness venues and facilities was alarmingly low, falling below 20%, which may hinder the promotion of active aging.

#### 3.2.3. Control Variables

Based on previous literature (Jiang & Yang, 2022), we controlled for a range of factors that may influence older adults’ mental health. These included age, gender, education, marital status, living arrangements, chronic diseases (e.g., hypertension and diabetes), the logarithm of annual income, the frequency of hospitalization among older adults within the past two years, and regional economic development. Additionally, we considered the *Hukou* based on the older adults’ household registration.

#### 3.2.4. Mediating Variable

To better understand how public sports facility availability in living communities affects mental health in older adults, this study considered physical activity and life satisfaction as mediating variables. Using CLASS data, physical activity was assessed from responses to the following question: “How often do you engage in physical activity?” Response options ranged from 1 to 5, encompassing categories including “less than one time per week”, “one time per week on average”, “two times per week on average”, “three times per week on average”, and “four or more times per week on average”. Life satisfaction was assessed from responses to the following question: “Overall, are you satisfied with your life at present?” Response options ranged from 1 to 5, encompassing categories including “very dissatisfied”, “somewhat dissatisfied”, “normal”, “somewhat satisfied”, and “very satisfied”. The detailed definitions of the aforementioned variables and measurements are presented in Table 1.

### 3.3. Analysis Strategy

Given that older people’s mental health is a continuous variable, we relied on the following ordinary least squares (OLS) model to investigate the relationship between public sports facility availability in living communities and older people’s mental health:

$${health}_{i}=\beta_{0}+\beta_{1}{facilities}_{i}+\beta_{2}{controls}_{i}+\varepsilon_{i}$$

where *β*_0_ is an intercept; *health_i_* represents the observed values of the explained variable, the mental health of older adults for individual *i*; *facilities_i_* represents the observed values of the explanatory variable, public sports facility availability in living communities for individual *i*; *controls_i_* is a set of control variables that may affect older adults’ mental health; *β_i_* denotes the corresponding estimated parameter for individual *i*; and *ε_i_* represents the random error term.

Although OLS can be used for estimating the effect of public sports facility availability in living communities on older adults’ mental health, concerns regarding the endogeneity issue of potential reverse causality remain. A common approach to resolve this problem is to use an instrumental variable (IV). Therefore, we employed the IV approach to address this endogeneity issue.

In this paper, the relief degree of land surface (RDLS) in China, calculated by You et al. (2018), was used as an IV to evaluate the influence of public sports facility availability in living communities on mental health among older adults^1^. Theoretically, the RDLS reflects the objective natural characteristics of a region. Flatter terrain facilitates the development of cities and infrastructure. Therefore, the RDLS is related to public sports facility availability in living communities. Regarding exogeneity, the RDLS is a macro-level variable that does not directly impact the mental health of older adults. Given that the RDLS satisfies the requirements of a valid IV—relevance and exogeneity—it is suitable for measuring the association between public sports facility availability in living communities and mental health among older adults.

Empirically, the results of the under-identification test (Kleibergen–Paap rk LM statistic was 145.171), weak-identification test (Cragg–Donald Wald F statistic was 127.529, and Kleibergen–Paap rk Wald F statistic was 186.412), and first-stage regression (F value was 186.410) indicated that our IV is valid. Overall, we considered the RDLS a reasonable IV. We used two-stage least squares (2SLS) to examine the endogeneity issue:

$$the\;first\;stage:\;{facilities}_{i}=\gamma_{0}+\gamma_{1}{IV}_{i}+\gamma_{2}{controls}_{i}+\theta_{i}\;the\;second\;stage:\;{health}_{i}=\lambda_{0}+\lambda_{1}{facilities}_{i}+\lambda_{2}{controls}_{i}+\mu_{i}$$

where *γ*_0_ and *λ*_0_ are intercepts; health, facilities, and controls have the same meanings as in the first equation; and *θ_i_* and *μ_i_* represent the random error terms. *IV* represents the instrumental variable, the RDLS in China.

Furthermore, to explore the influential mechanism and demonstrate support for Hypotheses 2a and 2b, the bootstrapping method was applied for mediation testing. The bootstrapping method involves repeatedly drawing samples with replacements from the original data and estimating the mediating effect for each resample. A significant mediating effect via physical activity or life satisfaction between public sports facility availability in living communities and older adults’ mental health was determined if the 95% confidence interval did not overlap zero (Alfons et al., 2022).

In addition, to demonstrate support for Hypotheses 3a, 3b, 3c, and 3d, we employed a moderation analysis by introducing an interaction term between public sports facility availability in living communities and a set of moderator variables. The moderation model is specified as follows:

$${health}_{i}=\alpha_{0}+\alpha_{1}{facilities}_{i}+\alpha_{2}{moderator}_{i}+\alpha_{3}{interaction}_{i}+\alpha_{4}{controls}_{i}+\delta_{i}$$

where *α*_0_ is the intercepts; *moderator_i_* refers to the moderating variable (age, income, education, and regional economic development); *interaction_i_* (*facilities_i_* × *moderator_i_*) captures the moderating effect; health, facilities, and controls have the same meanings as in the first equation; and *δ_i_* represents the random error terms. STATA 17.0 was used to perform the data analysis (StataCorp. LP., College Station, TX, USA).

## 4. Results

### 4.1. Descriptive Statistics

Table 1 presents the descriptive statistics for all the variables (*n* = 7811). Regarding the explained variable, the average score for older people’s mental health was 6.312 (SD = 3.055), indicating that their mental health condition was relatively positive. The average value of the explanatory variable, public sports facility availability in living communities, was 0.904 (SD = 0.946). This indicates that the overall availability of public sports facilities in living communities for older people was relatively low, with significant potential for improvement.

Regarding the mediating variables, the mean physical activity score was 3.970 (SD = 0.669), which corresponded to an average of three times per week. The mean life satisfaction score was 3.861 (SD = 0.781), which suggests that older people were somewhat satisfied with life. As for the control variables, the respondents’ mean age was 69.848 (SD = 7.308). A total of 51.6% of respondents were male. The average education score was 2.224 (SD = 0.720). Among the respondents, 73.4% were married, 37.4% were rural residents, 88.6% did not live alone, and 58.5% had a chronic disease. The mean logarithm of annual income was 8.975 (SD = 2.133). The mean value of hospitalization frequency among older people was 0.492 (SD = 1.153). The mean regional economic development value was 10.205 (SD = 0.619).

### 4.2. Benchmark Regression and Endogeneity Test

The results of the benchmark OLS regression and the IV-2SLS regression are shown in Table 2. As shown in the benchmark regression results, the coefficient for public sports facility availability in living communities was significantly negative (coefficient = −0.225; *p* < 0.01), suggesting that, after controlling for covariates, an increased availability of public sports facilities in living communities is associated with lower levels of depression, as well as better mental health, among older adults. After adding the IV, the coefficient for public sports facility availability in living communities was −2.283 (*p* < 0.01), further supporting the observed association. The results support Hypothesis 1.

### 4.3. Robustness Checks

#### 4.3.1. Changing the Sample Size

In this study, the dataset included older adults with a wide age range, with some individuals reaching advanced ages (103 years old). In the benchmark regression, we retained all observations to reflect the full distribution of the older population, avoiding arbitrary data exclusions. As physical function declines with age, older adults may face limitations in accessing and utilizing public sports facilities, which could in turn affect the potential health benefits they receive. To assess the robustness of our findings, we applied one-sided winsorization and truncation at the 5th percentile in order to account for the influence of data distribution on the estimation results. Table 3 presents the IV-2SLS regression results after applying these adjustments. The findings remain consistent with the benchmark regression, indicating that public sports facility availability in living communities is significantly associated with better mental health among older adults.

#### 4.3.2. Subgroup Robustness Check

To assess the robustness of the previous baseline regression results, this paper divided the sample into subsamples for group regressions. Table 4 presents the results of IV-2SLS regressions for six subgroups, categorized by gender, marital status, and chronic disease. The results indicate that regardless of whether the participants are male or female, married or otherwise, live with or without chronic diseases, public sports facility availability in living communities is consistently significantly associated with lower levels of depression and better mental health among older adults. This suggests that the effect of public sports facility availability in promoting mental health is consistent across various subgroups of older adults in China.

#### 4.3.3. Urban–Rural Heterogeneous Effect Analysis

Given the significant differences in infrastructure and institutional support across regions in China, it is plausible that the impact of public sports facility availability in living communities on mental health may vary across these contexts. To test the robustness of our benchmark finding and explore this potential heterogeneity, we divided older individuals into urban and rural groups to assess whether the effect of public sports facility availability in living communities differs across these groups.

**Figure 2:**
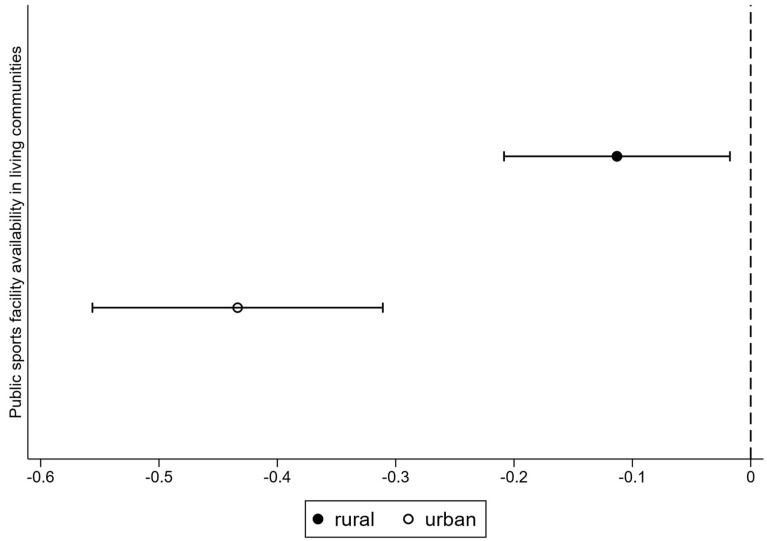
The heterogeneous effect of public sports facility availability in living communities on mental health among older people with different Hukou.

As shown in Figure 2, for both urban and rural older adults, the confidence intervals for the effect of public sports facility availability in living communities lie entirely to the left of the zero line, indicating that the positive association between public sports facility availability in living communities and older people’s mental health is significant across subgroups. At the same time, it clearly reveals a significant heterogeneity. The confidence intervals for the urban and rural subgroups do not overlap, and the point estimate for the urban group is significantly larger than for the rural group, suggesting that the positive influence of public sports facilities is significantly more pronounced in urban areas compared to rural ones.

### 4.4. Exploring the Mediating Roles of Physical Activity and Life Satisfaction

Theoretical analysis suggests that public sports facility availability in living communities is likely related to better mental health in older adults, potentially through increased physical activity frequency and enhanced life satisfaction. In this section, we explore the possible mediating mechanism, with the results shown in Table 5. This study employed the bootstrapping method to test the mediating role. For physical activity, the 95% confidence interval and the bias-corrected 95% confidence interval for the mediating effect were in the range [−0.009, −0.001] and [−0.010, −0.001], with neither overlapping zero. For life satisfaction, the results were in the range [−0.074, −0.043] and [−0.075, −0.044], with neither overlapping zero. These results suggest that physical activity and life satisfaction may serve as mediators in the association between public sports facility availability and mental health, supporting Hypotheses 2a and 2b.

### 4.5. Exploring the Moderating Effect of Age, Income, Education, and Regional Economic Development

**Figure 3:**
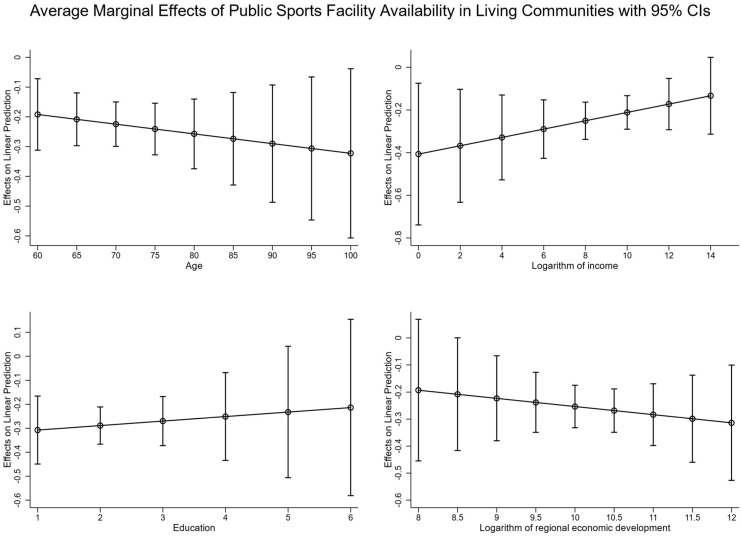
The average marginal effect of public sports facility availability in living communities on mental health among older people with different ages, income levels, educational backgrounds, and in areas with varying regional economic development.

Figure 3 presents the average marginal effects of public sports facility availability in living communities on mental health among older individuals with different ages, incomes, education levels, and in areas with varying regional economic development. The results suggest that age, income, education, and regional economic development moderate the relationship between public sports facility availability in living communities and older individuals’ mental health. Specifically, older age, lower educational attainment, lower income level, and higher economic development provinces show a stronger association with public sports facility availability in living communities and older people’s mental health. These results indicate that Hypotheses 3a, 3b, 3c, and 3d are supported.

## 5. Discussion

Mental health among older adults has become a serious public health concern due to the rapid progression of the aging population (Newman & Zainal, 2020). This study employed nationally representative data from 7811 individuals aged 60 and above to investigate the relationship between public sports facility availability in living communities and older adults’ mental health in China. We found that public sports facility availability in living communities (senior activity rooms, fitness venues/facilities, or outdoor activity areas) is significantly associated with better mental health in older individuals. Previous studies reached a similar conclusion, which suggests that the built environment (Weich et al., 2002; Araya et al., 2007; Berke et al., 2007; Thomas et al., 2007; Mair et al., 2008; Maas et al., 2009; Saarloos et al., 2011; Gong et al., 2016; Rautio et al., 2018; Domenech-Abella et al., 2020; Sokale et al., 2022; Mu et al., 2023; Wang et al., 2025) serves as an important factor in reducing depression among older adults, thus improving their mental health. As a key element of the built environment, public sports facilities are related to health-related behaviors and mental health outcomes in older people.

Furthermore, acknowledging the potential effects of individual characteristics on depression (McAneney et al., 2015), subgroup analyses were conducted based on gender, marital status, and chronic disease status. The findings consistently corroborated the benchmark results, affirming that public sports facility availability in living communities is significantly associated with better mental health among older adults. These results suggest robustness in our findings. Based on these associations, this result carries considerable implications for policymakers. First, efforts could be made to enhance public sports facility availability in living communities for older adults through strategic institutional arrangements. Second, efforts should be made to ensure that older adults have access to diverse and high-quality public sports facilities. This entails establishing well-equipped exercise venues that are conveniently located and designed to be age-friendly.

In addition to these general strategies, our findings on urban–rural disparities call for more targeted policy considerations. Our findings revealed that the positive association between public sports facilities in living communities is significantly stronger in urban areas. This disparity likely reflects the well-documented gap in the quantity, quality, and accessibility of public sports facilities in China. Therefore, beyond improving the availability of public sports facilities in living communities, promoting alternative forms of physical activity (e.g., organizing walking groups or community walks in culturally or historically significant sites) can further support rural older adults’ mental well-being.

Public sports facility availability in living communities may contribute to improving mental health among older adults by facilitating greater engagement in physical activity and improving their life satisfaction. Previous studies have highlighted the role of physical activity (O’Connor et al., 1993; Christmas & Andersen, 2000; Benedetti et al., 2008; Kandola et al., 2019; Pearce et al., 2022) and life satisfaction (Huebner et al., 2006; Bray & Gunnell, 2006; Headey et al., 1993; Soósová et al., 2021; Lin et al., 2024) in improving mental health among older adults. The empirical analysis results from the mediation test indicate that physical activity and life satisfaction serve as significant mediators in the relationship between public sports facility availability in living communities and mental health among older adults. These findings align with theoretical expectations. Public sports facilities within older adults’ living communities enhance their accessibility to exercise spaces. Moreover, providing free, publicly available facilities removes financial barriers, enabling older individuals to participate in physical activities without incurring additional costs (Steenhuis et al., 2009). Meanwhile, these facilities also serve as social hubs to foster social interaction and cohesion, both of which are key contributors to life satisfaction (Pfeiffer et al., 2020). Given these associations, public sports facility availability in living communities may be related to increased physical activity frequency and improved life satisfaction among older adults, which in turn are linked to mental health. Given the mediating role, policymakers should consider implementing community-based interventions, including health-oriented sports campaigns and organized fitness activities, to enhance participation rates in physical activity among older adults. Moreover, older adults should be encouraged to provide input on sports facility development through public consultations, citizen feedback channels, and government communication platforms. Ensuring the construction of age-friendly public sports facilities that enhance the life satisfaction of older persons should also be a priority.

In examining the moderating roles of age, income, education, and regional economic development, our findings suggest that advancing age, lower income, lower educational attainment, and increasing regional economic development are associated with a greater reliance of mental health among older adults on public sports facility availability in living communities. As aging progresses, older individuals may increasingly depend on community-based public sports facilities due to mobility constraints and reduced social networks (Yen et al., 2009; Lord et al., 2011). Consequently, it could be beneficial to improve age-friendly sports infrastructure within communities and schedule regular exercise programs to promote physical activity among older people. Older adults with lower incomes may be more dependent on free community-based public sports facilities due to financial limitations (Steenhuis et al., 2009; Withall et al., 2011). Hence, expanding free sports infrastructure and reducing access restrictions might help ensure older people’s participation. Individuals with lower education levels tend to have lower cognitive abilities and limited capacity to actively seek health-related information, increasing their dependence on accessible community sports facilities (Howard et al., 2006; Friis et al., 2016; Barrett & Riddell, 2019). Thus, alongside infrastructure development, it could be beneficial to implement tailored exercise guidance programs for older individuals. In provinces with higher economic development, governments allocate more resources to sports infrastructure, offering richer facility options for older people (Andreff, 2001; Burillo et al., 2011). To bridge regional disparities, we recommend considering increased sports investment and facility expansion in regions with lower economic development to support physical activity.

This study investigated the association between public sports facility availability in living communities and the mental health of older adults, including multiple robustness checks to ensure the reliability of the results. However, there is still room for further improvement. First, the 2014 CLASS databases do not include data on sports facilities and physical activity, and the 2018 and 2020 datasets are not publicly accessible. Thus, this study only used cross-sectional data from the CLASS 2016. Due to the cross-sectional nature of the data, the findings cannot support causal inferences but rather provide descriptive and correlational evidence on the relationship between public sports facility availability and mental health. Future research could leverage longitudinal datasets, when available, to investigate the long-term effects of public sports facility availability in living communities on mental health in older populations. Second, while the primary focus of this study was on the availability of public sports facilities in living communities, it is important to recognize that factors such as physical proximity, operational hours, and age-friendly infrastructure are equally critical. If relevant data become available, future research may explore these aspects to gain a fuller understanding of how public sports facilities affect the mental health of older people.

## 6. Conclusions

This study empirically examined the relationship between public sports facility availability in living communities and mental health among older adults in China. Using data from the CLASS 2016, this study found that public sports facility availability in living communities is significantly associated with a lower frequency of depressive symptoms, as well as better mental health, among older individuals, with this association remaining robust despite variations in sample size and subgroup. We also found clear differences between urban and rural areas, with urban older adults exhibiting a stronger influence compared to the rural older adults. Moreover, physical activity and life satisfaction serve as critical mechanisms through which public sports facility availability in living communities influences older people’s mental health. Age, income, education, and regional economic development are crucial in moderating the association between public sports facility availability and older adults’ mental health. These findings underscore the importance of policymakers in addressing the provision and accessibility of public sports facilities in living communities as a viable strategy for enhancing the mental health of older populations. Interventions should also aim to leverage the synergistic roles of physical activity and life satisfaction in order to maximize positive mental health outcomes.

## Figures and Tables

**Table 1:** Descriptive statistics (*n* = 7811).

Variable	Definition	Mean	Std. Dev.	Min	Max
Explained variable					
Mental health	Ranging from “0” to “18”. The lower the CES-D score, the better their mental health.	6.312	3.055	0	18
Explanatory variable					
Public sports facility availability in living communities	Ranging from “0” to “3”. The higher the score, the greater the availability of public sports facilities.	0.904	0.946	0	3
Mediating variable					
Physical activity	1 = less than one time per week; 2 = one time per week on average; 3 = two times per week on average; 4 = three times per week on average; 5 = four or more times per week on average	3.970	0.669	1	5
Life satisfaction	1 = very dissatisfied; 2 = somewhat dissatisfied; 3 = normal; 4 = somewhat satisfied; 5 = very satisfied	3.861	0.781	1	5
Control variables					
Age	Years old	69.848	7.308	60	103
Gender	1 = male; 0 = female	0.516	0.500	0	1
Education	1 = illiterate; 2 = primary school; 3 = junior high school; 4 = high school; 5 = junior college and above	2.224	0.720	1	5
Marital status	1 = married; 0 = otherwise	0.734	0.442	0	1
*Hukou*	1 = rural; 0 = urban	0.374	0.484	0	1
Living arrangement	1 = not living alone; 0 = living alone	0.886	0.318	0	1
Chronic diseases	1 = diagnosed; 0 = otherwise	0.585	0.493	0	1
Income	Logarithm of annual income	8.975	2.133	0	15.425
Hospitalization frequency	Times (within the past two years)	0.492	1.153	0	72
Regional economic development	Logarithm of regional Gross Domestic Product in 2016	10.205	0.619	7.853	11.300

**Table 2:** Results of benchmark regression (OLS and IV-2SLS).

Variables	(1)	(2)
	OLS	IV-2SLS
Public sports facility availability in living communities	−0.225 ***	−2.283 ***
	(0.037)	(0.304)
Age	0.023 ***	0.029 ***
	(0.005)	(0.006)
Gender	−0.026	−0.052
	(0.070)	(0.081)
Education	−0.290 ***	−0.070
	(0.050)	(0.066)
Marital status	−0.347 ***	−0.398 ***
	(0.093)	(0.107)
*Hukou*	0.356 ***	−0.695 ***
	(0.077)	(0.173)
Living arrangement	−0.732 ***	−0.705 ***
	(0.117)	(0.137)
Chronic diseases	0.570 ***	0.688 ***
	(0.070)	(0.084)
Income	−0.119 ***	0.001
	(0.019)	(0.028)
Hospitalization frequency	0.089 ***	0.053
	(0.032)	(0.036)
Regional economic development	−0.226 ***	0.191 **
	(0.055)	(0.094)
***Instrumental variable tests***		
Weak IV identification test	Kleibergen–Paap rk Wald F statistic	186.412 ***
	Cragg–Donald Wald F statistic	127.529 ***
Under-identification test	Kleibergen–Paap rk LM statistic	145.171 ***
First-stage regression	F value	186.410 ***
R^2^	0.074	—
n	7811	

Notes: Robust standard errors in parentheses; IV represents instrumental variable; *** *p* < 0.01, ** *p* < 0.05, and * *p* < 0.1.

**Table 3:** Robustness check after changing sample size (IV-2SLS).

Variables	(1)	(2)
	Winsorization	Truncation
Public sports facility availability in living communities	−2.283 ***	−2.482 ***
	(0.304)	(0.342)
Age	0.030 ***	0.025 ***
	(0.006)	(0.007)
Gender	−0.052	−0.058
	(0.081)	(0.086)
Education	−0.070	−0.060
	(0.066)	(0.071)
Marital status	−0.407 ***	−0.382 ***
	(0.107)	(0.114)
*Hukou*	−0.698 ***	−0.781 ***
	(0.173)	(0.188)
Living arrangement	−0.699 ***	−0.737 ***
	(0.137)	(0.148)
Chronic diseases	0.687 ***	0.701 ***
	(0.084)	(0.088)
Income	0.001	0.024
	(0.028)	(0.031)
Hospitalization frequency	0.052	0.030
	(0.036)	(0.037)
Regional economic development	0.193 **	0.245 **
	(0.094)	(0.101)
n	7811	7427

Notes: Robust standard errors in parentheses; *** *p* < 0.01, ** *p* < 0.05, and * *p* < 0.1.

**Table 4:** Results of subgroup robustness check (IV-2SLS).

Variables	(1)	(2)	(3)	(4)	(5)	(6)
	Male	Female	Married	Otherwise	Diagnosed Chronic Diseases	Otherwise
Public sports facility availability in living communities	−2.768 ***	−1.835 ***	−2.956 ***	−1.058 **	−1.159 ***	−5.659 ***
	(0.466)	(0.401)	(0.432)	(0.416)	(0.307)	(1.058)
Age	0.025 ***	0.031 ***	0.028 ***	0.025 ***	0.024 ***	0.018
	(0.009)	(0.008)	(0.008)	(0.009)	(0.007)	(0.015)
Gender			−0.028	−0.071	0.009	−0.155
			(0.103)	(0.150)	(0.094)	(0.196)
Education	0.038	−0.184 **	−0.030	−0.149	−0.144 *	0.153
	(0.099)	(0.090)	(0.085)	(0.117)	(0.077)	(0.168)
Marital status	−0.362 **	−0.393 ***			−0.480 ***	−0.699 **
	(0.184)	(0.130)			(0.122)	(0.299)
*Hukou*	−0.987 ***	−0.439 *	−0.999 ***	−0.140	−0.072	−1.966 ***
	(0.261)	(0.231)	(0.238)	(0.259)	(0.201)	(0.451)
Living arrangement	−0.718 ***	−0.753 ***	−0.446	−0.724 ***	−0.755 ***	−0.117
	(0.244)	(0.165)	(0.327)	(0.139)	(0.150)	(0.375)
Chronic diseases	0.770 ***	0.607 ***	0.708 ***	0.781 ***		
	(0.122)	(0.116)	(0.110)	(0.146)		
Income	0.030	−0.017	0.052	−0.094 **	−0.065 **	0.147 **
	(0.044)	(0.035)	(0.038)	(0.041)	(0.033)	(0.068)
Hospitalization frequency	0.066	0.045	0.037	0.071	0.062 *	0.160
	(0.051)	(0.048)	(0.046)	(0.054)	(0.037)	(0.128)
Regional economic development	0.441 ***	−0.066	0.409 ***	−0.251 *	−0.102	0.981 ***
	(0.140)	(0.126)	(0.128)	(0.136)	(0.102)	(0.273)
n	4030	3781	5734	2077	4566	3245

Notes: Robust standard errors in parentheses; *** *p* < 0.01, ** *p* < 0.05, and * *p* < 0.1.

**Table 5:** Results for mediating-effects analysis (bootstrapping 5000 times).

	Mediating Effect	Direct Effect
***Panel A: Physical activity***		
Normal-based [95% confidence interval]	[−0.009, −0.001]	[−0.293, −0.148]
*p* value	0.029	0.000
Bias-corrected [95% confidence interval]	[−0.010, −0.001]	[−0.292, −0.148]
***Panel B: Life satisfaction***		
Normal-based [95% confidence interval]	[−0.074, −0.043]	[−0.238, −0.095]
*p* value	0.000	0.000
Bias-corrected [95% confidence interval]	[−0.075, −0.044]	[−0.239, −0.096]
***Control variables***	Controlled	Controlled
n	7811	

## Data Availability

The original data presented in the study are openly available in the China Longitudinal Aging Social Survey (CLASS) at the following website: http://class.ruc.edu.cn/ accessed on 16 June 2023.

## Note

1 The RDLS for each region is derived from the disparity between maximum and minimum altitudes, terrain flatness, and the comprehensive area of the landform. Utilizing ArcGIS software and China’s 1:1,000,000 scale geographic digital elevation simulation dataset, this study extracts raster data at a spatial resolution of 1 km × 1 km and subsequently calculates the provincial-level RDLS.

## Appendix A

**Table A1:** Explanation of the assignments of mental health.

Variable	Description of the Assignment
Do you feel in a good mood?	“No” = 2; “Sometimes” = 1; “Often” = 0
Do you feel lonely?	“No” = 0; “Sometimes” = 1; “Often” = 2
Do you feel sad?	“No” = 0; “Sometimes” = 1; “Often” = 2
Do you feel like you are having a good time?	“No” = 2; “Sometimes” = 1; “Often” = 0
Don’t you feel like eating?	“No” = 0; “Sometimes” = 1; “Often” = 2
Are you having trouble sleeping?	“No” = 0; “Sometimes” = 1; “Often” = 2
Do you think you’re out of practice?	“No” = 0; “Sometimes” = 1; “Often” = 2
Do you feel you have nothing to do?	“No” = 0; “Sometimes” = 1; “Often” = 2
Do you find a lot of joy in life?	“No” = 2; “Sometimes” = 1; “Often” = 0
